# *Moringa oleifera*: An Updated Comprehensive Review of Its Pharmacological Activities, Ethnomedicinal, Phytopharmaceutical Formulation, Clinical, Phytochemical, and Toxicological Aspects

**DOI:** 10.3390/ijms24032098

**Published:** 2023-01-20

**Authors:** Ashutosh Pareek, Malvika Pant, Madan Mohan Gupta, Pushpa Kashania, Yashumati Ratan, Vivek Jain, Aaushi Pareek, Anil A. Chuturgoon

**Affiliations:** 1 Department of Pharmacy, Banasthali Vidyapith, Banasthali 304022, Rajasthan, India; 2 School of Pharmacy, Faculty of Medical Sciences, The University of the West Indies, St. Augustine 3303, Trinidad and Tobago; 3 Department of Pharmaceutical Sciences, Mohan Lal Sukhadia University, Udaipur 313001, Rajasthan, India; 4 Discipline of Medical Biochemistry, School of Laboratory Medicine and Medical Sciences, University of KwaZulu-Natal, Durban 4041, South Africa

**Keywords:** *Moringa oleifera*, traditional medicinal uses, pharmacological activity, phytochemistry, phytopharmaceutical formulation, toxicity

## Abstract

*Moringa oleifera*, also known as the “tree of life” or “miracle tree,” is classified as an important herbal plant due to its immense medicinal and non-medicinal benefits. Traditionally, the plant is used to cure wounds, pain, ulcers, liver disease, heart disease, cancer, and inflammation. This review aims to compile an analysis of worldwide research, pharmacological activities, phytochemical, toxicological, and ethnomedicinal updates of *Moringa oleifera* and also provide insight into its commercial and phytopharmaceutical applications with a motive to help further research. The scientific information on this plant was obtained from various sites and search engines such as Scopus, Pub Med, Science Direct, BMC, Google Scholar, and other scientific databases. Articles available in the English language have only been referred for review. The pharmacological studies confirm the hepatoprotective, cardioprotective, and anti-inflammatory potential of the extracts from the various plant parts. It was found that bioactive constituents are present in every part of the plant. So far, more than one hundred compounds from different parts of *Moringa oleifera* have been characterized, including alkaloids, flavonoids, anthraquinones, vitamins, glycosides, and terpenes. In addition, novel isolates such as muramoside A&B and niazimin A&B have been identified in the plant and have potent antioxidant, anticancer, antihypertensive, hepatoprotective, and nutritional effects. The traditional and nontraditional use of Moringa, its pharmacological effects and their phytopharmaceutical formulations, clinical studies, toxicity profile, and various other uses are recognized in the present review. However, several traditional uses have yet to be scientifically explored. Therefore, further studies are proposed to explore the mechanistic approach of the plant to identify and isolate active or synergistic compounds behind its therapeutic potential.

## 1. Introduction

*Moringa oleifera* (*M. oleifera*), the “miracle tree”, thrives globally in almost all tropical and subtropical regions, but it is believed to be native to Afghanistan, Bangladesh, India, and Pakistan [1]. The Moringa family comprises 13 species (*M. oleifera*, *M. arborea*, *M. rivae*, *M. ruspoliana*, *M. drouhardii*, *M. hildebrandtii*, *M. concanensis*, *M. borziana*, *M. longituba*, *M. pygmaea*, *M. ovalifolia*, *M. peregrina*, *M. stenopetala*), of which *M. oleifera* has become well known for its use in nutrition, biogas production, fertilizer, etc., [2,3]. Moringa has the unique property of tolerating drought [3]. Studies have shown that *M. oleifera* is among the cheapest and most reliable alternatives for good nutrition [4]. Nearly all parts of the tree are used for their essential nutrients. *M. oleifera* leaves have a high content of beta-carotene, minerals, calcium, and potassium [5]. Dried leaves have an oleic acid content of about 70%, which makes them suitable for making moisturizers [6]. The powdered leaves are used to make many beverages, of which “Zija” is the most popular in India [7]. The bark of the tree is considered very useful in the treatment of different disorders such as ulcers [8], toothache [9], and hypertension [10]. Roots, however, are found to have a role in the treatment of toothache [9], helminthiasis [11], and paralysis [12]. The flowers are used to treat ulcers, enlarged spleen, and to produce aphrodisiac substances [2]. The tree is believed to have incredible properties in treating malnutrition in infants and lactating mothers [3]. The present review aims to sum up the updated insight regarding the pharmacological activities, worldwide research analysis, toxicological, phytochemical, and ethnomedicinal properties of *M. oleifera*.

## 2. Material Method

### 2.1. Article Eligibility Criteria

In the framework of searching for study material, the following keywords were used: “*Moringa oleifera*”, “pharmacology *M. oleifera*”, “phytochemistry *M. oleifera*”, “ethnobotanical applications *M. oleifera*”, “toxicology *M. oleifera*”, and other combinations of terms such as biochemical constituents, taxonomic classification, geographical distribution, and plant formulation to search relevant peer-reviewed journals in various scientific databases such as Scopus, PubMed, Springer, Google scholar, and Wiley. Articles available in the English language have only been referred for review. Articles were analyzed by reading the title and abstracts of the articles found, which clearly indicated that they were all relevant.

### 2.2. Software and Techniques Used

Chemical structures identified in the plants were searched in the Webbook, Chemspider, and PubMed databases, and at the same time, the identified structures were drawn using Chem Draw (version 12.0.2). VOS Viewer software (1.6.18) was used to generate the map of global collaboration between countries. Global country boundaries (GIS layers) were obtained from an open-source web platform (DIVA-GIS) for geographic mapping. Spatial techniques were used with ArcGIS 10.1 to map *M. oleifera* indigenous and introduced countries and research activities in each country.

## 3. Worldwide Research and Collaboration

**Figure 1:**
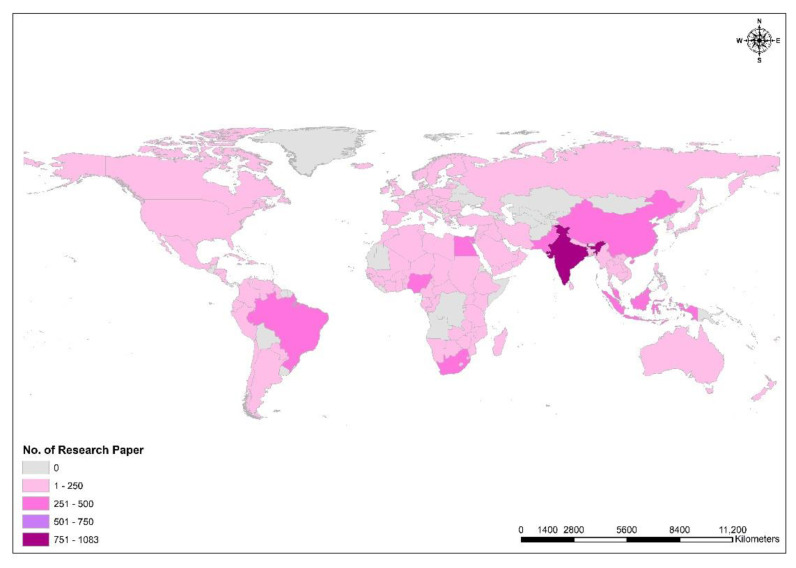
ArcGIS 10.1-based spatial distribution map highlights research papers published on *M. oleifera* worldwide. A spatial technique was used to generate a map, and GIS layers were obtained from DIVA-GIS, an open-source web platform.

A country-specific research database on *M. oleifera* was extracted from Scopus, and the data were linked directly to the country shapefile using the “connect field” function in the GIS environment for geospatial mapping. The analyses revealed that scientists from about 15 countries had published more than 100 research papers during the period 2000–2022. Among which India was the most prolific country (*n* = 1083), followed by Nigeria (*n* = 441), Brazil (*n* = 383), Egypt (*n* = 361), China (*n* = 331), Indonesia (*n* = 327), Pakistan (*n* = 281), South Africa (*n* = 272), Malaysia (*n* = 260), the United States (*n* = 214), Saudi Arabia (*n* = 205), Mexico (*n* = 163), Thailand (*n* = 127), and Italy (*n* = 105), (Figure 1).

**Figure 2:**
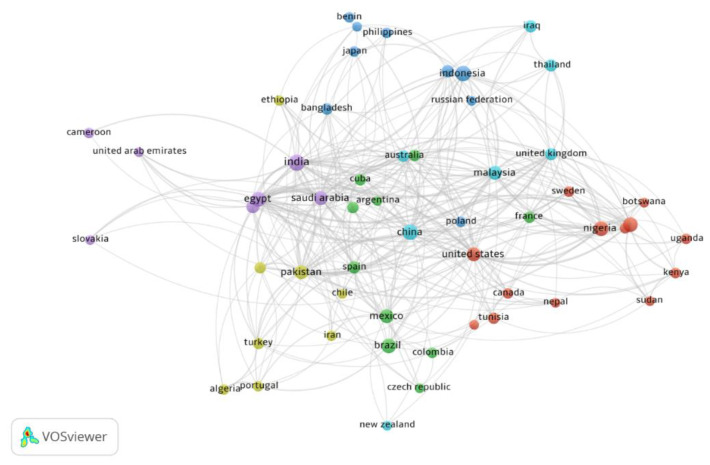
Network visualization of international collaborative research conducted for *M. oleifera* using VOS viewer (1.6.18).

International studies on *M. oleifera* involve the collaboration of scientists from countries such as India, China, Egypt, Saudi Arabia, Cuba, Australia, the United States, Nigeria, and Portugal, which is indicated by circles in the map (Figure 2). The analysis shows that many articles were published in the past decade, indicating the growing interest of researchers in this plant worldwide.

## 4. Taxonomical Classification

The plant *M. oleifera* belongs to the Kingdom: Plantae; Sub kingdom: Tracheobionta; Super division: Spermatophyta; Division: Magnoliophyta; Class: Magnoliopsida; Sub class: Dilleniidae; Order: Capparales; Family: Moringaceae; Genus: Moringa; Species: oleifera [3,13,14].

## 5. Morphology

The tree grows rapidly in loamy and well-drained sandy soils, preferring a height of 500 m above sea level [1]. Normally, the tree is small to medium in size, the leaves are naturally trifoliate, the flowers are born on an inflorescence 10–25 cm long [14], and the fruits are usually trifoliate and commonly referred to as “pods” [3]. The trunk usually grows straight but is occasionally poorly formed, the branches are usually disorganized, the canopy is umbrella-shaped; the brown seeds have a semi-permeable hull, and each tree has a capacity of about 15,000–25,000 seeds per year [10].

## 6. Botanical and Geographical Distribution

**Figure 3:**
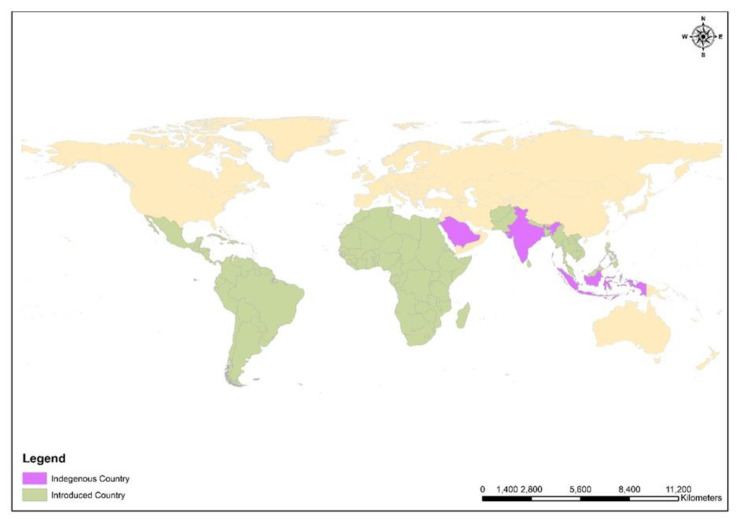
ArcGIS 10.1-based spatial distribution map of Moringa oleifera, the purple color shows the indigenous countries like India, Saudi Arabia, and East indies, whereas the green color shows the introduced countries and regions such as Tropical Asia, Latin America, Africa, Pacific Island, Caribbean Florida, Madagascar, Central America, Cuba, Philippines, Ethiopia, and Nigeria. GIS layers were obtained from DIVA-GIS, an open-source web platform.

*M. oleifera* is widely distributed worldwide, but its indigenous origin is in India, Arabia and the East Indies. It is common in Asia, Africa, the Caribbean, Latin America, the Pacific Islands, Florida, Madagascar, Central America, Cuba, the Philippines, Ethiopia, and Nigeria [2,15]. The history of the plant explains that *M. oleifera* was introduced from India to Africa, Southeast Africa, and the Philippines in ancient times [16,17] (Figure 3). It requires tropical and subtropical regions and grows at a temperature of about 25–35 °C [1]. *M. oleifera* is a deciduous type of tree typically grown in tropical and subtropical regions across the globe [18,19]. It grows best in indirect sunlight and without waterlogging, and the soil should be slightly acidic to alkaline. The tree begins to bear fruit at 6 to 8 months of age [18]. Commercially, it is grown in different countries such as Africa, Mexico, Hawaii, and South America, but due to different soil conditions, the nutrient content varies from country to country [3].

## 7. Ethnomedicinal/Traditional Properties

People worldwide have included *M. oleifera* in their diet since ancient times because of its vital therapeutic values (Table 1). Various medicines made from the plant are said to have ethnomedicinal properties for curing diseases and have been used for centuries. Approximately every part (leaf, pod, bark, gum, flower, seed, seed oil, and root) of this plant has been used to treat one disease or another [20]. Uses of *M. oleifera* are observed in pathological alterations such as antihypertensive [10], anti-anxiety [21], anti-diarrheal [22], and as a diuretic [23]. Moringa is also used to treat dysentery [24] and colitis [25]. A poultice made from Moringa leaves is a quick remedy for inflammatory conditions such as glandular inflammation, headache, and bronchitis [9]. The pods treat hepatitis and relieve joint pain [19]. The roots are conventionally used to treat kidney stones [26], liver diseases [27], inflammation [28], ulcers [29], and pain associated with the ear and tooth [30]. The bark of the stem is used to treat wounds and skin infections [31]. Indians use the gum extracted from this plant to treat fever, and it is also used to induce abortions [32]. The seeds of the plant act as a laxative and are used in the treatment of tumors, prostate, and bladder problems [33]. The seeds show promise for the treatment of arthritis by altering oxidative stress and reducing inflammation [34]. Preparations from the plant leaves benefit nursing mothers and malnourished infants and improve the general health of the population. The leaves have been useful for patients suffering from insomnia [35] and treating wounds [36]. Moringa is used incredibly extensively in the cosmetic industry nowadays, and in ancient Egyptian history, it was similarly used for preparing dermal ointments [37].

## 8. Pharmacological Uses

Recent pharmacological studies have revealed that different extracts of *M. oleifera* exhibit different pharmacological activities, such as antimicrobial [43], antifungal [44], anti-inflammatory [45], antioxidant [46], anticancer [47], fertility [48], wound healing [43], and other pharmacological activities mentioned below (Table 2).

### 8.1. Antimicrobial and Antifungal Activity

*M. oleifera* ethanolic root extract contains a compound N-benzylethyl thioformate (an aglycone of deoxyniazimincin) responsible for the antimicrobial and antifungal effect toward an extensive array of microbes and fungi [44]. *M. oleifera* methanolic leaf extract may exert inhibition of urinary tract infections caused by Gram-negative and Gram-positive bacteria such as *Klebsiella pneumoniae*, *Staphylococcus aureus*, *Escherichia coli*, *and Staphylococcus saprophyticus* [69].

The inhibitory effect of extracts from leaves, seeds, and stems of *M. oleifera* has been specified in various fungal strains such as Aspergillus flavus, Aspergillus terreus, Aspergillus nidulans, Rhizoctonia solani, Aspergillus niger, Aspergillus oryzae, Fusarium solani, Penicillium sclerotigenum, Cladosporium cladosporioides, Trichophyton mentagrophytes, Penicillium species, Pullarium species [44]. M. oleifera seeds have active components 4-(alpha-L-rhamanosyloxy) benzyl isothiocyanates, which are believed to be responsible for their antimicrobial activity [70]. The juice of Moringa leaves also showed potential against human pathogenic bacteria [43]. The methanolic leaf extract has nearly 99% inhibition against Botrytis cinerea (a necrotrophic plant fungus) [71].

The fruit of *M. oleifera* contains alkaloids, flavonoids, and steroids, which have an inhibitory effect against the culture of *Candida albicans* by either denaturing the protein or inhibiting the germination of spores through the steroid ring they contain [72].

Strong inhibitory effects of moringa seed kernel extract were observed for *Bacillus cereus*, *Staphylococcus aureus*, *Mucor species*, *and Aspergillus species*. However, it was less effective against *P. aeruginosa* and *E. coli*. This indicated that, except for *E. coli* and *P. aeruginosa*, moringa seed kernel extract might be utilized to treat infections caused by these species [73]. A recent study has been conducted, which states that only apolar extract obtained from seeds of *M. oleifera* showed anti-microbial activity against Gram-positive bacteria [74].

### 8.2. Anti-Inflammatory Activity

**Figure 4:**
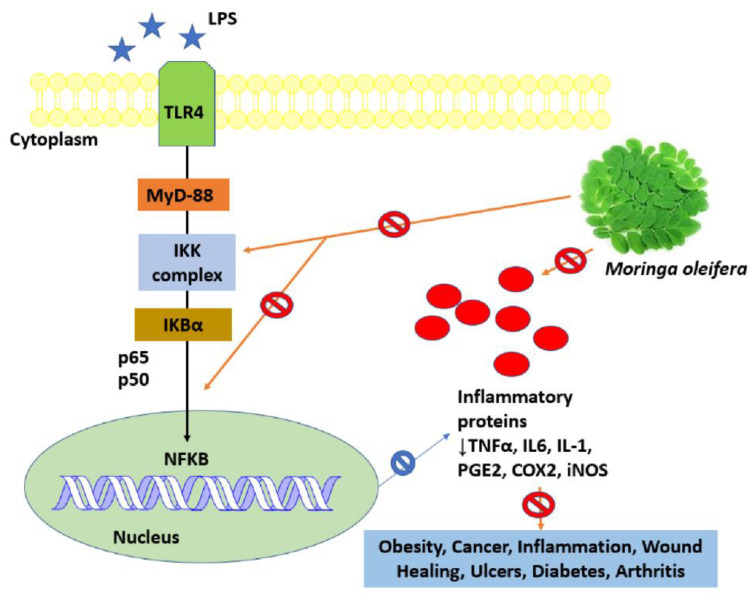
*M. oleifera*, as an oxidative and inflammatory marker, inhibits IKBα phosphorylation, thereby preventing NFKB (nuclear factor kappa B) inhibition. It prevents the nuclear translocation and dimerization of IkBα and NFKB, thereby inhibiting the formation of inflammatory proteins such as TNFα(tumor necrosis factor), COX-2(cyclooxygenase-2), IL6(interleukin -6), and iNos(inducible nitric oxide synthase) and thereby reducing the inflammation and curing other disorders like obesity, arthritis, cancer, diabetes, and ulcer.

A significant anti-inflammatory effect was observed in different parts of *M. oleifera* (leaf, pods, flowers, and roots). It was observed that the isolated compound (4-[2-o-Acetyl-alpha -l-rahamnoslyloxy) benzyl] thiocynate from Moringa possessed nitric oxide inhibitory activity and was subsequently found to be effective in Raw264.7 cell lines [75]. A compound derived from *M. oleifera* roots, known as aurnatiamide acetate and 1,3-dibenzylurea, inhibited TNF-α production [76]. Active compounds such as tannins, phenols, alkaloids, flavanoids, carotenoids β-sitosterol, vanillin, and moringin have anti-inflammatory properties [32]. The *M. oleifera* fruit extract blocked nuclear factor kappa B (NF κB) translocation, and the chloroform extract was found to be cytotoxic at high concentrations (500–1000 µg/mL) [77]. *M. oleifera* leaves extract was used in mice for treating atopic dermatitis in human keratinocytes and was found to be effective in reducing the expression of mannose receptor mRNA, thymic stromal lymphopoietin, and retinoic acid-related orphan receptor γT in ear tissues (Figure 4) [78].

### 8.3. Oxidative Stress

The results of *M. oleifera* were observed in methotrexate-induced mice. The study aimed to look into a probable palliative effect of *M. oleifera* extract on mice. The mice received the extract one week before administering methotrexate injection, and this treatment was continued for 12 days. The result showed that pretreatment with an extract of *M. oleifera* on mice poisoned with methotrexate could protect them from oxidative stress [79].

The antioxidant activity of ethanolic extract *M. oleifera* stems exhibited a protective effect against epidermal oxidative stress injury induced by H_2_O_2_ in keratinocytes. The result displayed that the stems showed antioxidant potential, and, therefore, can be used as an excellent and preventive source in animal epidermal oxidative stress injury [80].

The research investigated the antioxidant potential of Moringa leaves against diclofenac sodium-induced liver toxicity in animals. The researchers concluded that the extract was significantly effective against diclofenac-induced liver toxicity and, therefore, can be considered liver protective [81].

### 8.4. Anti-Oxidant Activity

Bioactive compounds such as glycosylates [82], isothiocyanates [62], thiocarbamates [83], flavonoids [84], and certain other compounds from Moringa pods have been investigated for reactive oxygen spices. The aqueous extract has been shown to be a potent free radical scavenger against free radicles [45]. Previous studies suggest that the antioxidant potential might be due to kaempferol, which is mainly found in plant leaves [43]. The synergistic outcome of Moringa was observed with piperine and curcumin on oxidative stress induced by beryllium toxicity in Wistar rats [85]. The alcoholic extract of the plant reduced glucose-induced cataractogenesis in isolated goat eye lenses by controlling GSH levels [86]. Myricetin, derived from the Moringa seed extract, has proved to be a better antioxidant than BHT (butylated hydroxytoluene) and alpha-tocopherol. *M. oleifera* leaf extract and compounds, such as isoquercetin, astragalin, and crypto-chlorogenic acid, help lower ROS in HEK-293 cells [87]. Moringa is also helpful in reducing plasma monoaldehyde (MDA) levels in fasting plasma glucose (FPG) concentration in healthy volunteers compared to the individuals fed with warm water. A dose-dependent upsurge in GSH and reduced MDA levels were observed with alcoholic extract of the plant without toxic effect till 100 mg/kg (Figure 4) [46].

### 8.5. Anti-Cancer Activity

Several parts of moringa (fruits, leaves, flowers, stems) have been shown to be beneficial against cancer, a deadly disease. The isolated compounds thiocarbamate and isothiocyanate from moringa act as inhibitors of tumor cells [15,47]. The dichloromethane fraction was found to be cytotoxic for MCF7 breast cancer cells [88]. Niazimincin has been projected as an effective chemopreventive agent in chemical carcinogenesis [44]. Alcoholic and hydro-methanolic extracts of fruits and leaves have shown significant tumor growth retardation in the melanoma mouse model [32]. Soluble cold distilled water from Moringa inhibited tumor cell growth and reduced ROS (reactive oxygen species) in cancer cells [45]. A recent study based on computational modelling suggests that M. oleifera contains rutin with the highest binding affinity with BRAC-1 (Breast Cancer Gene-1) [49].

### 8.6. Fertility and Anti-Fertility Activity

Adding to the list of beneficial effects of Moringa, the various parts of the plant possess fertility and abortion-inducing properties. The aqueous extract at a 200 and 400 mg/kg dose has been found to be more abortifacient and anti-fertility effects [44]. Recent studies on hot and cold extracts of leaves of *M. oleifera* propose that ingestion of Moringa before, after, and during pregnancy may lead to adverse fetal developmental outcomes by causing rigorous contraction of the uterine wall [89].

### 8.7. Hepatoprotective Activity

Among the numerous flavonoids (quercetin, kaempferol, isoquercetin, rhamnetin, etc.,) present in Moringa, quercetin in Moringa flowers is thought to be accountable for the hepatoprotective effect [44]. Methanolic extract at low dose showed changes in hepato-renal and hematological profile with significant changes in serum aminotransferase concentration, plasma cholesterol level, alkaline phosphate, bilirubin, and serum LPO levels. However, the higher dose of the extract altered total bilirubin, blood urea nitrogen, and non-protein nitrogen levels and decreased the clotting time [43]. Liver injuries induced by acetaminophen in Sprague-Dawley rats, where the standard drug taken was silymarin, Moringa showed similar hepatoprotective properties in these rats by dropping the levels of AST, ALT, and ALP [90]. The seeds were also found to be effective against carbon tetrachloride-induced liver fibrosis, as evidenced by a reduction in serum aminotransferase activity and globulin levels [91]. Treatment with this plant extract for about 21 days regularly as diet significantly reduced liver injury, and this effect was found due to alkaloid, quercetin, kaempferol, flavonoids, ascorbic acid, and benzyl glucosinolates in this plant [32].

### 8.8. Cardiovascular Activity

The freeze-dried aqueous and alcoholic extract of *M. oleifera* showed cardioprotective activity in animals induced with myocardial infarction by isoproterenol [92]. Chronic treatment of *M. oleifera* was effective on isoproterenol-induced hemodynamics and improved the levels of enzymes such as SOD, catalase, lactase dehydrogenase, glutathione peroxidase, and creatine kinase [92]. Butanolic extract has been proven to be a rich antioxidant source in rats with cardiac necrosis induced with isoproterenol. Moreover, it was found to significantly reduce inflammatory levels and myocardial necrosis due to the presence of the compound N-α-rhamnopyranosyl vincosamide [93]. Moringa leaves significantly lowered cholesterol levels by showing a protective effect on hypertensive rats. The active constituents believed to be responsible for this activity were identified as niazirmin A, niazirimin B, and niazimincin [32].

### 8.9. Anti-Ulcer/Gastroprotective Activity

Bisphenols and flavonoids found in moringa leaves showed a reduced level of ulcer index, duodenal ulcer, and stress ulcer in the ibuprofen-induced gastric ulcer model [32]. Moringa extract was shown to significantly reduce free radicals and neutralize the acidic behavior of gastric juice and have a protective effect on the development of gastric ulcer [94]. The presence of flavonoids in the plant has been shown to have a protective effect on ulcer formation by increasing capillary resistance and improving microcirculation, resulting in less cell injury [95].

### 8.10. Analgesic/Antipyretic Activity

Moringa leaf extract shows analgesic activity in almost all tree parts in central and peripheral animal models [32]. Multiple factions of alcoholic extracts such as petroleum ether, n-butanol, ethyl acetate, and dimethyl ether were found to have potent analgesic activity compared to standard aspirin [96]. At a dosage of 30–300 mg/kg, the polar and non-polar extract of leaves showed a remarkable drop in prostaglandin and bradykinin levels compared to the seed and root extract in a nociceptive study of formalin-induced paw edema [96]. The ether and ethyl acetate fractions of the seeds were studied in a hyperpyrexia model [97], keeping paracetamol as standard 200 mg/kg, and the extract proved to have the best antipyretic activity among all [45].

### 8.11. Neuropharmacological Activity

**Figure 5:**
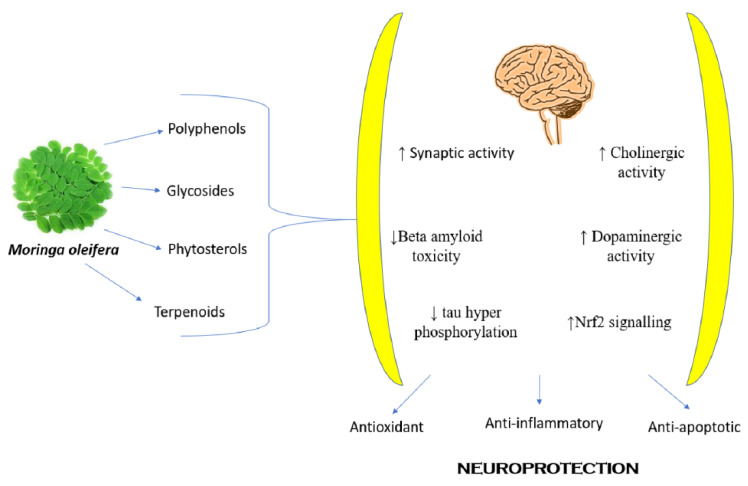
The various phytoconstituents present in *M oleifera* are responsible for numerous neuroprotective effects. *M. oleifera* is responsible for upregulating synaptic activity, cholinergic activity, dopaminergic activity, signaling of NrF2 (Nuclear factor erythroid 2-related factor 2), and simultaneously decreasing beta-amyloid toxicity and phosphorylation of tau proteins.

Previous results have proved that leaves extract reestablishes levels of monoamine in the brain and is very helpful in Alzheimer’s disease, while the in vitro activity of the ethanolic extract of the leaves showed an anticonvulsant effect on dopamine and norepinephrine levels, locomotor activity, and serotonin (5HT) in the brain in penicillin-induced convulsions [98,99]. The methanolic root extract in mice induced by pentobarbital sodium and diazepam has remarkable sedative effects on the CNS by improving sleep duration [35]. The toluene acetate fraction of the methanolic extract proved its potency as a possible nootropic agent [97]. The leaves have shown good anticonvulsant activity in a phenyl tetrazoline and maximal electric shock-induced model using male albino mice [98]. The aqueous extract of the roots blocked the epileptic seizures induced by penicillin in adult albino rats [99]. The ethanolic leaves extract exhibited anxiolytic properties, which were confirmed in behavioral experiments using the actophotometer and the rotarod device, respectively (Figure 5) [32].

### 8.12. Neuropathic Pain

The broad spectrum of phytoconstituents of the leaves extract of Moringa has led researchers to develop a herbal alternative for treating chronic neuropathic pain caused by constriction. The need to limit conventional analgesics for this disease. Diabetic rats inflicted with neuropathic pain caused by chronic constriction were used for the study. Tests conducted before and after treatment with moringa leaves showed that they significantly altered the neuropathic pain condition in diabetic rats. It suggests that the drop in oxidative stress might be the underlying mechanism in treating neuropathic pain and thus could be used as an effective novel source for the same [100].

A research team explored the bio-guided fractions of Moringa seed extract on a diabetes-induced neuropathic pain model. After conducting various oxidative and other experimental studies on induced and treated rats, the team concluded that the extract-treated rats exhibited reasonable glycemic control and antinociceptive properties and proved to be a powerful neuroprotective agent with a high margin of safety (Figure 5) [101].

### 8.13. Wound Healing Effect

A significant effect in studies on wound healing after incision or excision was demonstrated for ethyl acetate, and water extract of *M. oleifera* leaves at a 300 mg/kg dose [43]. Studies reported that in preclinical studies, leaves, seeds, and dried pulp extracts have shown effective enhancement of wound closure, granuloma rupture strength, and reduction of skin rupture strength in the scar area [102]. Leaf extracts have shown promising results in diabetic animals by improving the downregulation of inflammatory markers and increasing the vascular endothelial growth factor level in the injured tissue [32]. Compounds present in aqueous extract have shown a considerable effect on diabetic foot ulcers by downregulating the levels of various inflammatory markers [102]. The researcher conducted an in vitro assay to select the standardized extract with the highest potency, which was then converted into a film for wound healing. The result showed that the aqueous extract had the maximum cell proliferation and migration properties among the different extracts [103].

### 8.14. Immunomodulatory Activity

Methanolic extract of the plant contains active constituents such as isothiocyanate and glycoside cyanide, which exhibit immunostimulatory activity and effectively enhance immunity. The recent review paper suggests that various bioactive compounds have been used to treat various immune-related disorders such as cancer, hypertension, and diabetes, thereby enhancing host immunity [104].

### 8.15. Hematological Activity

*M. oleifera* has demonstrated its significant benefits in hematological activities. A randomized, double-blind study suggests that aqueous leaf extract effectively improves women’s low hemoglobin levels (8–12 g/dL) [105]. Another study showed that *M. oleifera* leaves, when taken for 14 days by healthy volunteers, significantly improved platelet counts [32].

### 8.16. Anti-Obesity Activity

**Figure 6:**
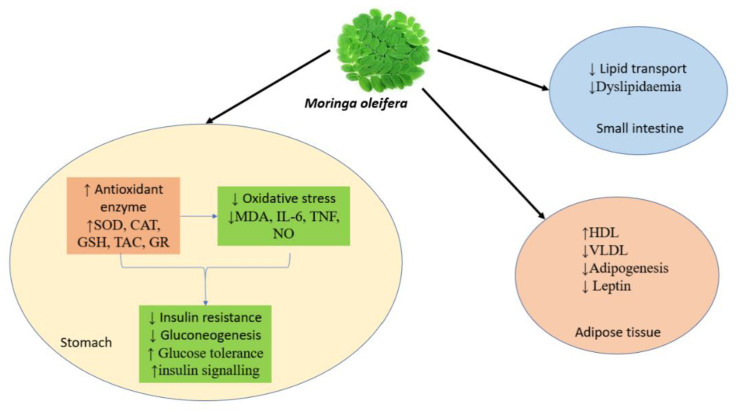
*M. oleifera* as a promising anti-obesity agent. Various in-vitro findings suggest that supplements of *M. oleifera* cause direct inhibition of pancreatic lipase, thus reducing the conversion of triglycerides into simple. Moringa has fat storage regulation by upregulation of lipolysis-associated protein and down-regulating the expression of protein related to fat storage. It is also effective in the improvement of antioxidant levels. Besides these, Moringa is also responsible for increasing ghrelin levels and decreasing leptin, producing a feeling of satiety.

In a study, oral treatment with leaf powder of *M. oleifera* for a duration of nearly 49 days was found to significantly reduce body mass index (BMI) in rats suffering from hypercholesterolemia [106]. The mechanistic approach behind this was the downregulation of mRNA expression of the hormones resistin and leptin and the concomitant increase in regulation of the gene adiponectin in rats [32]. A recent study revealed the mechanistic approach for the anti-obesity effect of *M. oleifera*. The plant significantly improved lipid profile by reducing body weight. It also regulated adipogenesis-related genes, increased glucose tolerance, and decreased levels of hormones such as vaspin, leptin, and resistin (Figure 6) [107].

### 8.17. Anti-Allergic Activity

The ethanolic seeds extract reduced histamine release and also suppressed the anaphylaxis induced by anti-immunoglobulin G. The mechanism underlying this effect may be the membrane-stabilizing potential of mast cells in an oval albumin sensitization model [32].

### 8.18. Anti-Diabetic Activity

Moringa leaves showed excellent results in the glucose tolerance of Wistar and Goto-Kakizaki rats and also lowered blood glucose levels. The aqueous extract showed an antidiabetic effect in rats by controlling blood glucose levels, protein, sugar, and hemoglobin [45]. The leaves of the plant were found to lower glucose levels within three hours of intake, but not more than the standard drug glibenclamide. Moringa seeds, when administered orally, contain insulin-like proteins that have antigenic epitopes such as insulin and exhibit antihyperglycemic activity [108]. Leaf extracts of the plant also have antidiabetic activity as they increased CAT and MDA levels, reduced FPG levels, hemoglobin levels, LDL-C, and VLDL-C in type 2 diabetic patients and, most importantly, increased insulin levels in healthy subjects [109]. The seed extract of the plant reduced LPO levels and amplified the antioxidant effect in mice induced with streptozotocin, the seed extract was also able to reduce IgG, IgA, and IL -6 parameters and pancreatic β-cell activity, and it was suggested that the bioactive compound responsible for this effect were quercetin, kaempferol, glucomoringin, chlorogenic acid, and isothiocyanates [110].

### 8.19. Diuretic Activity

The alcoholic and aqueous root extract of *M. oleifera* significantly affects calcium oxalate urolithiasis in male rats. This reduction was observed due to the decrease in the retention level of oxalates, calcium and phosphates as well as serum urea nitrogen, creatinine, and uric acid [23].

### 8.20. Angiotensin Converting Enzyme (ACE) Activity

Compounds such as niazimin-A, niazicin-A, and niaziminin-B are stated to be present in the *M. oleifera* plant extract. These compounds were found to have potent antihypertensive activity when targeted to (ACE), an important enzyme of the renin-angiotensin system. The researchers observed this activity by protein–ligand docking and found that the compounds have a high affinity for the angiotensin-converting enzyme compared with captopril and enalapril (standard drug) [111]. The angiotensin enzyme rennin plays a prominent role in regulating blood pressure and leading to diseases such as hypertension, kidney disease, and other cardiovascular diseases. The study found that the role of *M. oleifera* with two other plants (*Azadirachta indica* and *Hibiscus sabdariffa*) inhibited the enzyme with percentage inhibition (71.8%, 74%, and 73.4%) compared to standard drugs (captopril and enalapril). The compound responsible for this activity of Moringa was termed β-sitosterol [112].

### 8.21. Anti-Venom Effect

The leaves of the plant extract have been shown to be effective against the venom of *Naja Nigricollis* (a snake species) in rats. This snake’s venom contains potent neurotoxins that cause the degradation of phospholipids at the plasma membrane, affecting the normal neurotransmission process and causing hemolysis and hemorrhage. The results showed that Moringa extract effectively cured acute anemia, and a remarkable increase in micronucleated polychromatic erythrocytes was observed in rats treated with *M. oleifera* [113].

### 8.22. Cytotoxicity Effect

The cytotoxic potential of *M. oleifera* on human mesenchymal myeloma cell lines is observed in methanolic extract. The results of the extracts showed a higher ID50 value than other extracts. The researchers also found that the alkaloids and flavonoids contained in the plant showed some similarity to vincristine and vinblastine by random experiments. Therefore, the plant can be recommended for the herbal treatment of myeloma patients [47].

It was found that the ethanolic leaves extract of *M. oleifera* contains active constituents that can alleviate cyclophosphamide-induced testicular toxicity by promoting genes associated with the functional integrity of spermatozoa and enlargement of DNA in spermatogonia. Therefore, the administration of the extract not only improved blood and intestinal hormone levels but also modulated the expression of genes responsible for Sertoli and spermatogonial cells [114].

## 9. Toxicity

Various experimental procedures were conducted to evaluate the toxic potential of the plant. A random selection of female non-pregnant Wistar albino rats was conducted with an oral dose of 2000 mg/kg aqueous methanol solution. Blood samples were collected, and the ALT, AST, and total bilirubin content were determined. The outcome of the study suggested that the lethal dose of the aqueous extract was higher than 2000 mg/kg in female rats [53].

A similar study was also conducted in Sprague-Dawley rats to evaluate the acute toxic potential of Moringa leaf powder. The experiment also found that oral administration of dried leaves up to 2000 mg/kg had no harmful or lethal effect on the human body [115].

The toxicity of *M. oleifera* seeds in rats was observed at acute and subacute levels (methanolic extract). Acute toxicity was seen at a dose of 4000 mg/kg, whereas mortality was observed at a dose of 5000 mg/kg. Therefore, in a nut shell, it could be summarized that the seed extract could be safe for nutritional use [116].

Experiments conducted for acute and subacute studies indicated that the stem bark extract did not cause any toxic effect in the acute and subacute toxic studies up to 2000 mg/kg. Therefore, the researchers concluded that the stem bark of *M. oleifera* can be considered non-toxic when administered orally [117].

The subacute toxicity test at a dose of 250, 500, and 1500 mg/kg was performed for 60 days. The result showed that the lethal dose was 1585 mg/kg without significant alterations in sperm quality, biochemical, and hematological parameters compared to the control group [105].

The acute toxic study (5000 mg/kg) and subacute (40–1000 mg/kg) results showed no adverse reaction during these studies. However, increased ALT, ALP, and lower creatinine levels were observed. Therefore, it could be concluded that consumption is safe, but intake should not surpass 70 gm/day to prevent cumulative toxicity [118].

## 10. Clinical Trials

To date, 25 clinical studies have been conducted on *M. oleifera*, fifteen of which have been completed. Nine of these fifteen studies addressed *M. oleifera* as part of a diet, while the remaining studies were limited to disease-specific drug interventions. Overall, the studies demonstrated the efficacy of using moringa for conditions such as malnutrition, chronic kidney disease, HIV infection, and reproductive health [119].

A clinical study highlighted the significant role of *M. oleifera* as an anti-asthmatic agent. In this study, researchers used seed kernels of *M. oleifera* to treat symptoms of bronchial asthma. Candidates were selected based on inclusion and exclusion criteria, with respiratory parameters and blood samples recorded before and at the end of three weeks of treatment with *M. oleifera*. The extract was administered to patients in the form of dried powder at a dose of 3 g twice daily for three weeks, and they were instructed to take water with the powder. Symptoms were graded as severe, moderate, and mild on a point table. The results indicate that symptoms and respiratory functions decreased; *M. oleifera* seeds can effectively treat bronchial asthma [120].

## 11. Phytopharmaceutical Formulations

Plant extracts have always attracted researchers’ attention for producing various pharmaceutical products. This process usually involves the production of medicinal products characterized by two things: first, the production of a stable product, and second, patient compliance. The advantage of Moringa plant extracts is that it appears to be exceedingly safe at the doses and in the amounts commonly utilized for therapeutic efficacy [20]. *M. oleifera* has been widely accepted in the research area, and scientists have used an array of approaches to develop various formulations. The various phytoformulations prepared using *M. oleifera* are tabulated below (Table 3).

## 12. Miscellaneous Uses

A study was performed on *M. oleifera* using the HPLC-based cyclo condensation method. Astragalin and isothiocyanates were used as markers for standardization of the plant. The conclusive result of the study suggests that the standardized method might be useful for assessing the quality of the development of cosmetic and natural health products [144].

The extract of *M. oleifera* leaves was helpful in eliminating the adverse effects of neem oil, which is used in aquaculture as an insecticide to control predators and parasites of fish fry. The researchers concluded that the extract of *M. oleifera* leaves eliminated the oxidative stress and toxicity caused by neem oil [145].

The lower yield of okra (Abelmoschus esculentus) was studied. The low production of these crops was due to infestation by pests and insects and poor soil nutrient content. In order to improve production conditions, different chemical pesticides were used, which brought further environmental risks. The use of *M. oleifera* aqueous leaf extract at different concentrations (1:30 and 1:40) proved beneficial for okra [146].

The efficacy of *M. oleifera* leaf and root extract was evaluated as a plant growth regulator and biopesticide in the wheat harvest. The researcher used different concentrations (5, 10, 12.5, 25% *w*/*w*, *w*/*v*, *v*/*v*) of Moringa leaf and root extract at different stages of the wheat plant. Significant plant growth was observed, resulting in increased yield and a decrease in aphid invasion [147].

*M. oleifera* is rich in macronutrients and micronutrients, vitamins, phytohormones, alkaloids, and flavonoids, which make this plant a multipurpose plant. Recent research has shown that Moringa extract is also helpful in tolerance to abiotic and biotic stress under stressful environmental conditions [148].

The therapeutic effect of bioactive constituents (flavonoids, alkaloids, tannins, isothyocyanin and beta-sitosterol) present in *M. oleifera* has been reported in chronic diseases such as hyperlipidemia [149], hypertension [150], hepatoprotective [151], anti-cancer [152], Alzheimer’s disease [153], Parkinson’s disease [154].

The combined effect of *M. oleifera* and praziquantel in rats was studied by a group of researchers. The seeds and leaves of *M. oleifera* were considered to evaluate their bioavailability with praziquantel and also the in vivo effects of the same were observed on Taenia crassiceps. The study showed that the combined action of both had a significant amount of cytocidal activity compared to the rats, which were only administered with praziquantel [155].

A variety of bioactive nutrients, such as flavonoids and vitamins, is available in the *M. oleifera* plant. The in vitro study conducted by a group of researchers focused on bioactive compounds that make up the nutritional potential of plants [156]. The conclusive statement of the researcher revealed that the high content of proteins, lipids, and sulfur-containing amino acids and the relative lack of toxic components make moringa a great nutritional alternative for humans [157].

The bioactive isolate palmitic acid from the leaf extract of Moringa has been attributed to broad therapeutic benefits. A team of researchers studied this isolate against a wide range of microbial and fungal strains. The results showed that it had the highest zone of inhibition for both fungal and microbial strains [4].

The polyphenols and flavonoids present in *M. oleifera* to scavenge free radicals could be useful in developing an anticancer drug delivery system. Nanoparticle technology was used to incorporate Moringa extract as a drug carrier. Treatment of HeLa cell lines with a single dose of the plant showed that the composite film of the plant extract was efficient in killing malignant cells compared to other isolated and purified phytocomponents on the market [158].

The bioactive components of *M. oleifera* inhibit the inflammatory markers in lipopolysaccharide-induced human macrophages. The induced macrophages were treated with M. oleifera extract. Thereafter the treated cells were tested for their anti-inflammatory and cellular mechanism. The results revealed that the extract suppressed mRNA expression of IL -6, IL -1, NF-κB (P50), PTGS2, and TNF-α. At the same time, the inhibition of phosphorylation of IκB-α and nuclear factor (NF)-κB was also observed in the study. The researchers’ final statement suggests that the blocking of NF-κB and IκB-α may be the reason for the inhibition of inflammation [159].

Apart from its wide use in preventing and curing various human diseases, Moringa is known for a number of non-medicinal uses, chief among which is its use for poultry, especially in curing viral infections (Newcastle Disease Virus) and other parasitic and bacterial diseases that cause mortality in animals [160]. The plant also serves as an important growth promoter for farmers in the production of tomatoes, peanuts, corn, and wheat in their early vegetative stages [161]. Environmentally friendly biopesticides are produced from this plant, which is cheap and easily available and helps in curing various plant diseases [162]. Studies have shown that the total crop production increased by 20–35% by using *M. oleifera* leaf extract, which is a good sign for increasing agricultural growth at a minimal cost [163]. The aqueous extract of *M. oleifera* is a source of various minerals and growth promoters (indole acetic acid, gibberellins, cytokines). It thus can be used as an effective plant biostimulant that could be a simple alternative to the artificial fertilizers and pesticides available in the market [161]. The methanolic extract of the plant is found to be rich in potassium, calcium, carotenoid, phenols, and zeatin, and when three sprays of this extract are applied on the oilseed rape plant, it is observed that the pods, twigs, height, and number of seeds increase significantly compared to the untreated control group [164]. The ability of the plant to resist drought is due to the plant hormone “zeatin”, which is present in large quantities in the methanolic extract, so the plants exposed to such climatic conditions when sprayed with the methanolic extract of Moringa showed improved growth characteristics compared with well-watered plants [165]. The tree is efficient in removing water hardness and is used by African tribes as a cheap source compared to chemical softeners [166]. A study conducted by several groups found that treating river water in African countries with Moringa seeds reduced color and microorganisms by 90% and microorganism (*Escherichia coli*) levels by up to 95%. Previous reports indicated that a water sample treated with *M. oleifera* seeds reduced the hardness content of the water by 50–70%, which used to be 80.3 g L^−1^ CaCO3 [167]. It has also been shown to be an effective solution for treating turbidity, alkalinity, and dissolved organic carbon. It is suggested that Moringa could be, to some extent, an alternative to chemical alum used to remove water turbidity [168]. Moringa is a good source for curing plant diseases and can be a good option for biopesticides [162]. Since various plant pathogens affect the plants, Pythium debaryanum-a pathogen responsible for damping-off disease-can be cured by adding leaves to the soil [169]. Nearly all plant part (fruits, flowers, leaves, seeds, roots) is believed to have different properties that can heal the body spiritually and psychologically [34].

## 13. Phytochemistry

Almost all parts of *M. oleifera* and its isolates have been studied for research. Based on the literature collected between 2010 and 2022, more than 90 compounds from the genus Moringa have been identified, many of which have therapeutic potential. The isolates fall into the category of proteins and amino acids [170], phenolic acids [171], carotenoids [172], alkaloids [173], glucosinolates [174], flavonoids [175], sterols [175], terpenes [176], tannins and saponins [177], fatty acids [178], glycosides [179], and polysaccharides [180] (Table 4 and Table 5).

The leaves contain larger amounts of calcium, potassium, proteins, and amino acids such as arginine and histidine [170].

*M. oleifera* leaves contain a higher concentration of phenolic acids and flavonoids, including cinnamic acid, sinapic acid, syringic acid, gentisic acid, gallic acid, ferulic acid, protocatechuic acid, vanillin, caffeic acid, o- coumaric acid, p-coumaric acid and epicatechin are phenolic acids. In contrast, quercetin, catechin, myricetin, and kaempferol fall under the category of flavonoids having excellent therapeutic activity and are listed above in Table 2 [171,175,177].

The carotenoid lutein is present in larger quantities in the leaves of *M. oleifera* [172]. The therapeutic efficacy of the plant is due to the active constituents present in the plant leaves. The leaves of the plant revealed nearly thirty-five compounds by gas chromatography–mass spectrometry. Among the thirty-five isolated compounds, some important ones isolated in the leaves were palmitoyl chloride, cis-vaccenic acid, 5-O-acetyl-thio-octyl, pregna-7-dien-3-ol-20-one, γ-sitosterol, β-l-rhamnofuranoside, and tetradecanoic acid [178].

Two new alkaloids, marumoside A and marumoside B, were also isolated from *M. oleifera* leaves; another type of alkaloid, aurnatiamide acetate was isolated from *M. oleifera* roots [173,178]. Moringin and moringinine are the two alkaloids present in the plant’s stem [179].

*M. oleifera* is a rich source of glucosinolates. The most abundant glucosinolate present in the species is 4-O-(α-L-rhamnopyranosyloxy)-benzyl glucosinolates, also known as glucomoringin [174].

A sterol isolate, β-sitosterol was found in seeds and leaves of *M. oleifera* [173]. Another type of sterol glycoside β-sitosterol-3-O-β-D-galactopyranoside was extracted from *M. oleifera* bark [174,175].

An interesting category of diterpenes and terpenes is found in *M. oleifera* leaves. Among which Phytol is diterpene alcohol, a major component of chlorophyll, and is found abundantly in leaves of the plant. At the same time, terpenes and their derivatives are present in trace amounts (linalool oxide, farnsylacetone, isolongifolene α-ionene, and α- and β-ionone), whereas hexahydro-farnesyl acetone can be found in abundance [176].

Seed oil extract of *M. oleifera* commonly contains tannins and saponins, which are responsible for numerous pharmacological activities [177].

Fatty acids such as arachidic acid, octacosanoic acid, oleic acid, palmitic acid, stearic acid, linolenic acid, behenic acid, and paullinic acid are found in *M. oleifera* seeds [178].

Two glycosides, namely niazirin and niazirinin were extracted from the ethanolic extract of *M. oleifera* [179].

**Figure 7:**
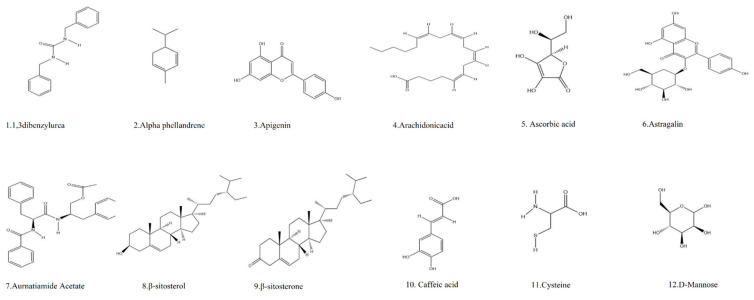

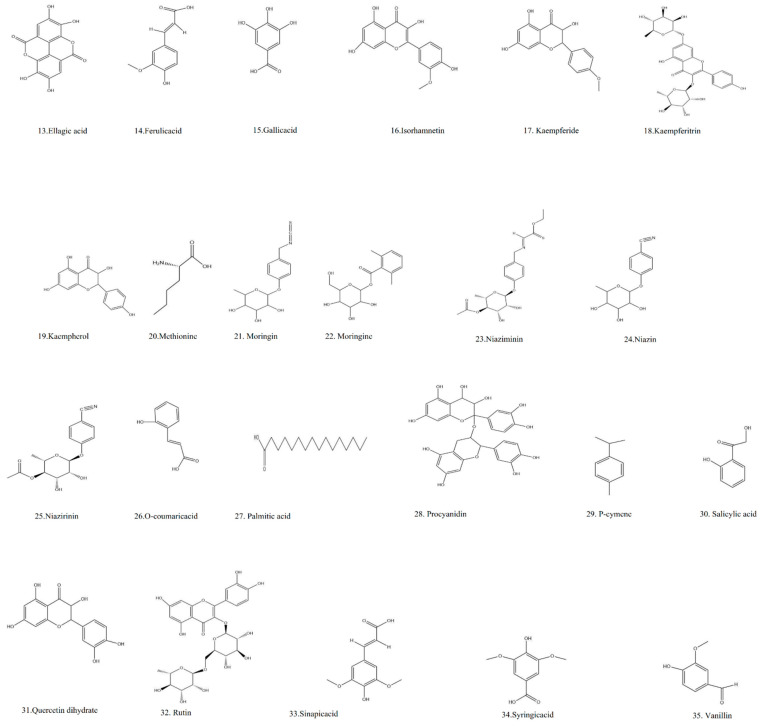
Structure of some key phytoconstituents isolated from *M. oleifera*.

The exudate of the gum contains a large number of poly saccharides such as D-galactose, L-arabinose, D-xylose, L-rhamnose, D-mannose, and -glucuronic acid [180]. The structure of some of the key phytoconstituents isolated from *M. oleifera* is shown in Figure 7.

## 14. Current Status

Moringa is a versatile plant with numerous benefits, and the current status of the plant suggests that it can be used in various pharmacological activities and their related formulations, biomedical applications, livestock, poultry, and fish production, enormously. Extensive research conducted in India, Nigeria, Brazil, and China during 2019–2022 has created a valuable resource for researchers worldwide. After an extensive study of this plant, it was found that *M. oleifera* has evolved to benefit humans in many ways. A large number of nutrients and phytoconstituents in this plant make it suitable for consumption by humans and animals. Due to its high anti-oxidant properties, it has become a pharmaceutical option for the production of formulations such as wound healing, anti-cancer, and anti-ageing etc. It is suitable not only for human use but also as a fertilizer in various forms extracted from *M. oleifera*. Besides its benefits, it also has severe toxic and abortifacient effects when taken in large quantities.

## 15. Conclusions and Future Perspective

The review summarizes various aspects of *M. oleifera*, including its worldwide research, ethnopharmacology, pharmacology activities, phytochemistry, phytopharmaceutical formulations, clinical studies, toxicology, and other miscellaneous parameters. The presence of alkaloids, phenolic acid, glycosides, sterols, glucosinolates, flavonoids, terpenes and fatty acids are responsible for the medicinal effects of *M. oleifera*. In addition, *M. oleifera* is also rich in compounds such as vitamins, micronutrients, and carotenoids which increase its medicinal value and consumption as a superfood. Pharmacological studies show that the active constituents of the plant have effectively cured various diseases such as neuropathic pain, cancer, hypertension, diabetes, obesity etc. Nevertheless, several phytochemicals have yet to be explored for their possible therapeutic benefits. In addition to its clinical use, the plant is also used as an effective biostimulant for farmers in their fields and has proven to be a cost-effective alternative. A literature survey suggested that much preclinical research has been carried out in the last few years. In the future, more clinical studies are required to investigate the efficacy of the plant in life-threatening diseases such as coronavirus outbreaks, an acquired immunodeficiency syndrome (AIDS), and various cancers. Moreover, further mechanism base studies are also proposed to explore the mechanistic approach of the plant to identify and isolate active or synergistic compounds. Overall, *M. oleifera* signifies its name, “Miracle tree,” and appears to be a phytopharmaceutical and functional food that, if consumed daily, can potentially treat various chronic diseases in humans and could be used by medical practitioners as a safer alternative to treat various ailments.

## Figures and Tables

**Table 1:** Uses of Moringa oleifera listed in Ayurvedic medicinal textbook.

Name of Ayurvedic Text	Form of Plant Used	Treatment	References
Charaka Samhita (1000 BC- 4th Cent. AD)	Powder Decoction	Used for treatment of worms and headache, Ascites, edema Hiccough and asthma, deafness, tinnitus in the ear, worm’s manifestation.	[38]
Ashtanga Hridaya (7th Cent. AD)	Oil	Ear ache, deafness, and tinnitus in the ear	[39]
Kashyapa Samhita (6–7th Cent AD)	Decoction Oil	Puerperal disorder, sleeplessness Edema	[40]
Sharangadhara Samhita (13 Cent. AD)	Decoction	Conjunctivitis	[41]
Yogaratnakara (17th Cent. A.D.)	Decoction	Enlargement of spleen, worm edema, Ascites, fever, abscess.	[42]

**Table 2:** Phytoconstituents of Moringa and their relevant therapeutic effects.

**Plant Part**	**Compound**	**Class**	**Structure**	**Therapeutic Activity**	**References**
Leaves	Rutin (555.6 µg/g)	Flavonoid	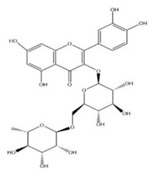	Found to have maximum affinity and interaction towards BRAC-1 gene.	[49,50]
Leaves	Kaempferol (197.6 µg/g)	Flavonoid	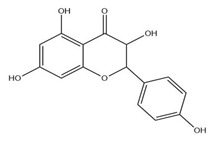	Oxidative damage protective activity.	[51]
Leaves	Quercetin (2030.9 µmol/100 g)	Flavonoid	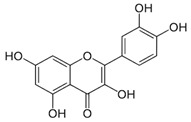	Exerts an excellent effect as anti-diabetic agent.	[52]
Leaves	O coumaric acid (0.536 mg/g)	Phenolic acid	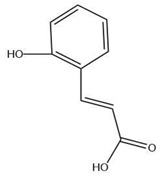	Antioxidant and anti-microbial	[53,54]
Leaves	Myricetin (5.804 mg/g)	Flavonoid	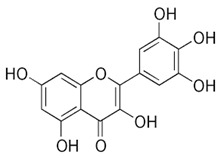	Potential prevention of diabetes mellitus and other diabetic complications	[54]
Leaves	Ellagic acid (0.078 to 0.128 mg/g)	Polyphenol	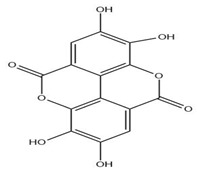	Prevents viral and bacterial infections, potential antioxidant	[54,55]
Leaves	Ferulic acid (0.078 to 0.128 mg/g)	Phenol	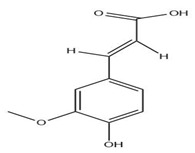	Promising results as anti- cancer, antioxidant, antithrombotic, anti-arrhythmic, and anti-inflammatory.	[54,56]
Leaves	Caffeic acid (0.409 mg/g)	Phenol	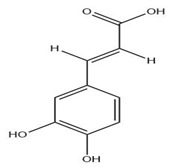	Boosts athletic performance, reduces fatigue, helps weight loss, protects against herpes, HIV, cancer.	[54,57]
Leaves	Sinapic acid (trace amount)	Phenol	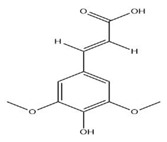	Cardioprotective, renoprotective, anxiolytic, neuroprotective.	[54,58]
Leaves	Gallic acid (1.034 mg/g)	Phenol	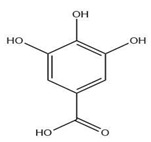	Anti-inflammatory, anti-neoplastic, anti-oxidant	[54,59]
Leaves	Syringic acid (trace amount)	Phenol	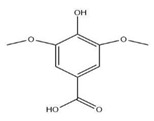	Anti-oxidant, antimicrobial.	[54,60]
Leaves	Isorhamnetin (0.118 mg/g)	Flavonoid	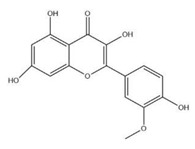	Anti-oxidant	[54,61]
Seeds	Myricetin (5.804 mg/g)	Flavonoid	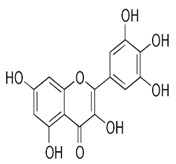	Potential prevention of diabetes mellitus and other diabetic complications	[54]
Seeds	Glucomoringin	Glucosinolates	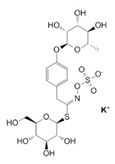	Anti-inflammatory, pain relieving, anti-oxidant, antihypertensive.	[62]
Seeds	β-sitosterol	Phytosterol	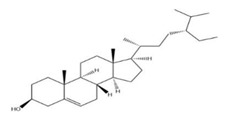	Anti-inflammatory	[63]
Seeds	Arachidic acid	Fatty acid	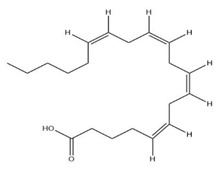	Increased breast milk production	[64]
Seeds	Oleic acid (70% *w*/*w*)	Fatty acid	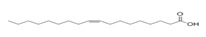	Reduces blood pressure and reduces free radical damage to the cell.	[65]
Seeds	Myristic acid	Fatty acid	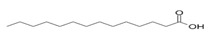	Anxiolytic effect, used in membrane localization of the enzyme.	[66]
Seeds	Palmitic acid	Fatty acid	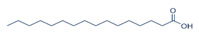	Trypanocidal and anti-leukemic effect	[67]
Seeds	Procyaniadin	Flavonoid	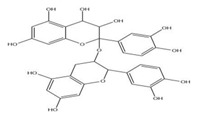	Cardioprotective	[68]
Flower	D-mannose	Carbohydrate	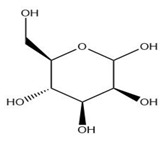	Treatment of deficiency caused by genetic defects, and acute urinary tract infections.	[69]
Stem	β-sitosterol	Phytosterol	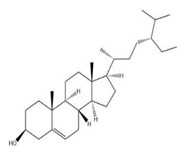	Anti-oxidant, cardiovascular, immunomodulatory	[63]

**Table 3:** Phytopharmaceutical formulations prepared using *M. oleifera* extract.

Plant Part Used	Nature of Extract	Formulation	Method of Preparation and Polymers/Excipients Used	Application	Inference	References
Leaves	Ethyl acetate	Polyherbal formulation	Suspending method (carboxy methyl cellulose)	Anti-ulcer	The potent anti-ulcer activity was observed in polyherbal formulations.	[121]
Leaves	Aqueous/methanolic	Polyherbal ointments	Water in oil mixing (wool fat, hard paraffin, cetostearyl alcohol, PEG4000, PEG400, sorbitol mono-oleate, liquid paraffin, white beeswax, span 60, tween 60)	Edema	The methanolic extract had a more anti-inflammatory effect than the aqueous extract. Apart from potent anti-inflammatory activity, the -miscible base ointment showed good drug diffusion.	[122]
Seed	Oil	Micro-dispersion	Vortexing (Span 80, tween 80)	Anti-inflammatory	Showed higher permeation rather than pure oil. Tween 80 enhances the permeation.	[123]
Leaves	Ethanolic	Lozenges	Wet granulation (Polyvinyl-pyrrolidone, magnesium stearate, menthol, vanillin)	Anti-microbial activity	Flavoring agents gave better acceptance for the consumer than formulation with no addition of flavoring agent. Due to limited solubility of quercetin in aq. phase, only 40–50% of quercetin is released.	[124]
Seed	Oil	Nano-micelle	Microemulsion method (Tween 80, Ethanol)	Mitochondrial cancer cell apoptosis	Targetability increases when seed oil is formulated in nano-micelle. Nanomicelles kill 50% colon cancerous Caco-2 cells compared to seed oil which kills only 40% of cancer cells.	[125]
Leaves + fruits (*Embelia ribes*)	Hydro-alcoholic	Thermo-reversible in-situ nasal gel	Cold method (poly (ethylene glycol) (PEG400), Pluronic F127, xanthum gum, carbopol 934), hydroxypropyl methylcellulose (HPMC K4M).	Allergic rhinitis	On increasing PF127 concentration, the viscosity of in-situ gel increases. PF127 exhibited less mucoadhesion as compared to HPMC/carbopol/xanthan gum. Plant in situ gel was formulated to overcome low bioavailability and first-pass metabolism.	[126]
Leaves	Aqueous, ethanolic	Film dressing	Solvent casting method (Alginate, pectin)	Wound healing	Aq. leaf extracts showed effective results in cell proliferation and migration properties. Optimal physicochemical properties were exhibited by the extract.	[127]
Leaves	Ethanolic	Effervescent tablets	Wet granulation (70% ethanol, lactose, citric acid, tartaric acid, sodium bicarbonate, aspartame, PEG600)	Anti-anemia	The effervescent times of all formulations were less than 2 min, and thus all lie within the range mentioned in the pharmacopoeia standard.	[128]
Seed	Oil	Anti-inflammatory cream	Triturating process (Oleic acid, sodium hydroxide, potassium hydroxide, aluminum hydroxide, liquid ammonia, sodium benzoate).	Anti-inflammatory	Optimized formulation showed 60% inhibition, whereas the standard inhibited 65% protein denaturation. Oil reduced paw edema by 64%, whereas formulation reduced it by 70%.	[129]
Leaves		Silver NPs (AgNPs)	Shaking method (Silver nitrate)	Anti-fungal activity	ZOI increases with an increase in the concentration of AgNPs against Candida albicans. The agar plate distributed with the highest concentration of AgNPs exhibited lowest cultural population.	[130]
Leaves	Aqueous	Hydrocolloid film dressing	Solvent casting method (sodium alginate, pectin)	Wound healing in diabetic condition	Hydrocolloid dressing is free of any irritants or toxic substances. 0.5% and 0.1 % film dressing converts to gel after being exposed to wounds compared to 1% due to rapid gelation.	[131]
Leaves	Hydro-alcoholic	In-situ gel	Cold technique (Pluronic F127, gellan gum, glycerine, Carbopol 934)	Allergic rhinitis	Dose-dependent results of the prepared formulation were seen in mice induced with ova albumin along with a significant decrease in Ig E levels.	[132]
Leaves	Aqueous	Nanofibers impregnated onto Hydrocolloid film	Electrospinning (poly-(ethylene oxide) (PEO), sodium alginate, pectin, glycerol)	Chronic Wound dressing	PEO concentrations and nanofiber diameter were directly proportional. Polymers in low concentration lead to poor entanglement of chain and produce beads or lumps of nanofibers and also form discontinuous fibers. At very high concentrations, the electrospinning stainless needle gets blocked, and polymer ejection gets obstructed. Electrospinning time increases, and the release of bioactive from film increases.	[133]
Leaves	Aqueous/Ethanolic	Silver nanoparticle loaded Composites	Sodium hypophosphite, silver nitrate, citric acid, Kaolin (clay), Chitosan (LMW), sodium carbonate.	Anti-oxidant	Ethanolic extract proved to be a better composite as compared to aqueous extract. ZOI is higher with alcoholic extract than the aqueous extract. Fabrics with AgNPs composites showed excellent UV protection than clay composites.	[134]
Leaves	Hydro-alcoholic	Polymeric microparticles (MPs)	Spray dried method (Chitosan)	Exuding wound treatment	Decreasing the extract amount increases the entrapment efficiency. Unloaded MPs have wrinkled surfaces, whereas extract-loaded MPs have spherical and smooth surface. The gel produces a covering layer that protects the wound because of chitosan.	[135]
Leaves	Aqueous	Iron oxide nanorods	Mixing method (Iron (III) chloride hexahydrate)	Anti-bacterial property	Nanoparticles formed showed concentration-dependent inhibition. Iron nanorods showed strong inhibition towards bacterial strain compared to conventional anti-bacterial drugs.	[136]
Seed	Oil	Suppositories	Pour molding method (Macrogol, dika fat, liquid paraffin, Polyethylene glycol 1000 & 4000, petroleum ether)	Hemorrhoids	Suppositories containing Moringa seed oil showed effective results against hemorrhoids. The incorporation of seed oil in suppositories reduced their melting point.	[137]
Leaves	Ethanolic	Oral suspension	Stirring method (Sodium carboxymethyl cellulose, propylene glycol, benzoate, sorbitol)	Hepato-protection against Isoniazid	Viscosity increases as the concentration of suspending material increases.	[138]
Leaves	Ethanolic	Granules	Wet granulation method (Gum Arabic, HPMC, Methocel K100M CR, magnesium stearate, Avicel PH200, tween 20, 40, 80, span 20, 40, Poloxamer 407, sodium lauryl sulphate)	Anti-inflammatory and anti-arthritic	Tween 20 improved dissolution as compared to extract. Granule formation increases the solubility of *M. oleifera* extract, thereby effectively reducing paw thickness. Body weight reduced at first six days and later increased significantly due to gum arabica.	[139]
Leaves	Powder	Chewable gummy tablets (CGT)	Heating and Congealing (Gelatin, high methoxyl pectin, mannitol, sucrose, propylene glycol, citric acid, corn oil, sodium benzoate)	Evaluation of Chewable gummy tablets	Pectin produced CGT with a higher swelling ratio compared to gelatin. Higher pectin and gelatin concentrations strengthened the gel structure, and the dissolution time increased CGT. Pectin-based CGT’s did not show syneresis, whereas CGT’s with 5% gelatin experienced syneresis. Higher hardness values are due to increased gelatin concentration. Pectin-based CGT had higher gumminess.	[140]
Seeds	n-hexane	Herbal hydrogel	Mixing method (Carbopol, propylparaben sodium, methylparaben sodium, propylene glycol, triethanolamine)	Wound healing	*M. oleifera* hydrogel showed significant wound-healing activity. Hydrogel positively affects the proliferation of cells, granular tissue formation, and epithelization.	[141]
Leaves	Aqueous	Phytosome	Thin-layer hydration method (soy phosphatidylcholine, TrizolTM)	Breast cancer	A dose less than 2000 mg/kg showed better in vivo assessment. Inhibition of 4T1 cells is dose-dependent.	[142]
Leaves	Ethanolic	Emulgel	Dissolving method (Carbopol 940, triethanolamine Tween 80)	Anti-oxidant activity	After eight weeks of storage, decreased in the pH of preparation as Moringa leaves experience decomposition of fatty acid.	[143]

**Table 4:** Phytochemicals present in different plant part extracts of *M. oleifera*.

S. No		Extract	Methanolic Roots	Ethanolic Roots	Methanolic Leaves	Ethanolic Leaves	Aqueous Leaves	Methanolic Seeds	Ethanolic Seeds	Aqueous Seeds
	Chemical									
1.	Alkaloid		+	+	+	+	+	+	+	+
2.	Tannins		+	+	+	+	+	+	-	+
3.	Flavonoids		+	+	+	+	+	-	+	-
4.	Saponins		+	+	+	+	+	-	+	-
5.	Terpenoids		+	+	+	+	+	-	-	-
6.	Glycosides		+	+	+	+	+	-	-	-
7.	Steroids		+	+	+	+	+	-	-	-
8.	Coumarins		-	-	+	+	-	-	-	-
9.	Proteins		-	-	-	-	-	+	+	-
10.	Starch		+	+	-	-	-	-	-	-

**Table 5:** Select phytoconstituents of *M. oleifera* isolated through various techniques.

Constituents	Concentrations #	Category	Technique Used	Reference
LEAVES				
Isoquercetin	1575.28 μg/g (*w*/*w*)	Flavonoids	UPLC-ESI-MS/MS	[181]
Astragalin	0.153 μg/g (*w*/*w*)	Flavonoids	HPLC	[182]
Isorhamnetin	2.9 mg/g (*w*/*w*)	Flavonoids	HPLC	[183]
Daidzein	ND *	Flavonoid	HPLC	[183]
Apigenin	ND *	Aglycone	HPLC	[184]
Luteolin	ND *	Flavonoid	HPLC	[184]
Genistein	ND *	Flavonoid	HPLC	[184]
4-(α-L-rhamnopyranosyloxy) benzyl glucosinolate	33.9 mg/g	Glucosinolates	LC/MS	[184]
4-[(2′ -O-acetyl-α-L-rhamnosyloxy) benzyl] Glucosinolate	21.84 to 59.4 mg/g	Glucosinolates	HPLC	[184]
Epicatechin	5.68 mg/g	Flavonoid	HPLC	[184]
Ferulic acid	0.078 mg/g	Phenolic acid	HPLC	[184]
Caffeic acid	0.409 mg/g	Phenolic acid	HPLC	[184]
Ellagic acid	0.018 mg/g	Phenolic acid	HPLC & MS/MS	[185]
Sinalbin	2.36 mg/g	Glucosinolates	HPLC	[185]
Sinapic acid	ND	Phenolic acid	HPLC &MS/MS	[185]
Chlorogenic acid	0.018 mg/g	Phenolic acid	HPLC &MS/MS	[185]
Gallic acid	1.034 mg/g	Phenolic acid	HPLC &MS/MS	[186]
Salicylic acid	0.14 μg/g	Phenolic acid	HPLC GC MS	[186]
Vicenin-2	193.43 ng/mg	Flavonoid	HPLC PDA	[187]
Quercetin-3-O-(6′′-malonyl) glucoside	ND *	Flavonoid	HPLC DAD	[188]
Pyrrolemarumine-4′′ -O-α-Lrhamnopyranoside	NA ^	Pyrole alkaloid	NMR	[189]
4-[(4′ -O-Acetyl-α-L-rhamnosyloxy)benzyl]	2.16 to 5.0 mg/g	Glucosinolates	HPLC	[190]
**SEEDS**				
Niazimicin	NA ^	Isothiocyanates	HPLC	[173]
Niazirin	NA ^	Isothiocyanates	HPLC	[173]
**ROOTS**				
Arachidic acid	ND *	Fatty acid	GC-MS	[190]
**BARK**				
β-Sitosterol-3-O-β-D-galactopyranoside	26.67 mg/g	Glucoside	HPTLC	[190]

ND—not detected; NA—not available; * lower but appreciable amount present; ^ compounds were isolated, but data not available; # the concentration of the constituent may vary according to geographical location.

## Data Availability

Not applicable.
